# Oleanolic Acid Slows Down Aging Through IGF-1 Affecting the PI3K/AKT/mTOR Signaling Pathway

**DOI:** 10.3390/molecules30030740

**Published:** 2025-02-06

**Authors:** Yan Xu, Jianlei Wei, Wang Wang, Zebin Mao, Didi Wang, Tao Zhang, Pengxia Zhang

**Affiliations:** 1 Medical College of Basic Sciences, Jiamusi University, Jiamusi 154000, China; xzh15247664359@163.com (Y.X.); wjl17686266799@163.com (J.W.); wang13836405335@163.com (W.W.); wangdidi@jmsu.edu.cn (D.W.); 2 Key Laboratory of Microecology-Immune Regulatory Network and Related Diseases of Heilongjiang Province, Jiamusi University, Jiamusi 154000, China; 3 Department of Biochemistry and Molecular Biology, Health Science Center, Peking University, Beijing 100191, China; zbmao@bjmu.edu.cn

**Keywords:** Oleanolic acid, Insulin-like growth factor-1, Aging, PI3K/AKT/mTOR

## Abstract

Objective: A pentacyclic triterpene, oleanolic acid (OA), has anti-inflammatory activity. The role of oleanolic acid in aging is poorly understood, and the regulatory mechanism of IGF-1 signaling in aging is still not fully understood. Thus, we hypothesized that OA could delay aging by regulating the PI3K/AKT/mTOR pathway via insulin-like growth factor-1 (IGF-1). Method: This study initially established a replicative aging model and a bleomycin-induced aging model in human dermal fibroblast (HDF) and mouse embryonic fibroblast (MEF) cell lines. On this basis, IGF-1 inhibitors or IGF-1 recombinant proteins were then combined with OA (at a concentration of 20 μM) and treated for 72 h. The project plans to detect the expression of aging-related proteins such as CDKN2A (p16) using Western blot technology, detect the expression of aging-related factors such as Interleukin-1 beta (IL-1β), Interleukin-6 (IL-6), and Interleukin-8 (IL-8) using Real-Time Quantitative Polymerase Chain Reaction (RT-qPCR), Enzyme-Linked Immunosorbent Assay (ELISA), and other technologies, and combine Senescence-Associated β-Galactosidase (SA-β-gal) staining to detect changes in aging. Results: The expression of IGF-1, PI3K/AKT/mTOR, aging-related proteins P16, and aging-related secretory factors (SASP) IL-1β, IL-6, and IL-8 was increased in senescent cells. After treatment with jujuboside, the expression of IGF-1, PI3K/AKT/mTOR, aging-related protein P16, and aging-related secretory factors IL-1β, IL-6, and IL-8 were decreased. Conclusion: The findings suggested that OA slowed down aging by inhibiting the PI3K/AKT/mTOR expression through IGF-1. These findings suggest OA as a potential new drug and its mechanisms for anti-aging.

## 1. Introduction

Aging is a normal process that involves many factors. According to research, inflammation is one of the causes of aging. As the body gets older, senescence-related secretory factors and aging-related proteins increase. Therefore, reducing senescence-related secretory factors and senescence-related proteins may delay aging. Several risk factors will speed this process leading to disease, including diabetes, cardiovascular disease, neurodegenerative disorders, and cancer [1,2,3,4]. Senescence is a biological process that is the gradual and inevitable decline of cellular function and structure, and it is marked by a series of irreversible processes [5,6,7,8]. It begins with a halt in the normal course of growth and development, where cells cease to divide and expand at an ever-increasing rate. As this happens, tissues gradually shrink, organs become less efficient, and overall physiological functions are compromised. Although the biological basis of aging is far from understood, researchers have suggested that targeting the aging process itself can improve the outcomes of many age-related pathologies [9,10]. There is evidence that insulin-like growth factors (IGF) and insulin signaling regulate aging in worms, insects, and mammals [11,12]. Studies in mice have shown that heterozygous deletion of the IGF-1 receptor prolongs lifespan [13], while another study has shown that by caloric restriction, lowering IGF-1 levels prolongs lifespan in monkeys [14]. In addition, significant prolongation of longevity was detected in both sexes in mice lacking growth hormone (GH) or GH receptors, in which circulating IGF-1 levels were significantly suppressed. However, the specific role of IGF-1 in the aging process has not been fully elucidated [15]. GH acts on GH receptors in hepatocytes, thereby stimulating the secretion of IGFs, particularly IGF-1, which activates the downstream PI3K-AKT and MTORC1 networks via IGF-1R to activate trophic signaling, thereby promoting growth and development. In a variety of model organisms, spontaneous pathways or engineered mutant pathways can extend lifespan and delay the emergence of age-related deterioration [16]. The mammalian/mechanistic target of rapamycin (mTOR) is a key component of cellular metabolism that integrates nutrient sensing with cellular processes to promote cell growth and proliferation. Furthermore, mTOR was shown to be upregulated in replicative senescence that mediates the aging process [17]. In the present study, we observed that IGF-1 could inhibit mTOR expression and delay cellular senescence.

Oleanolic acid (OA) is a pentacyclic triterpenoid rich in plants and has important physiological functions [18]. Previous studies by our research group have found that OA is rich in traditional Chinese medicine extracts and it has significant anti-tumor effects and curative effects on a variety of diseases [19,20]. As a new antiviral drug, OA has been widely used in the clinical treatment of acute and chronic hepatitis [21]. OA is a new type of lipid-lowering drug used to reduce low-density lipoprotein (LDL) and triglyceride (TG). Therefore, OA can effectively improve the occurrence of cardiovascular diseases and reduce the occurrence of stroke [22]. Our previous study also found that OA has an obvious protective effect on intestinal mucosal injury caused by 5-FU, and its mechanism may be related to its anti-inflammatory effect [23]. However, whether OA exerts an anti-aging effect by regulating IGF-1 and the PI3K/AKT/mTOR pathway has not been reported. Based on the previous work, this study intends to further study the regulatory effect of OA on IGF-1 and the PI3K/AKT/mTOR signaling pathway.

## 2. Results

### 2.1. IGF-1 Was Highly Expressed in Senescent Cells

**Figure 1:**
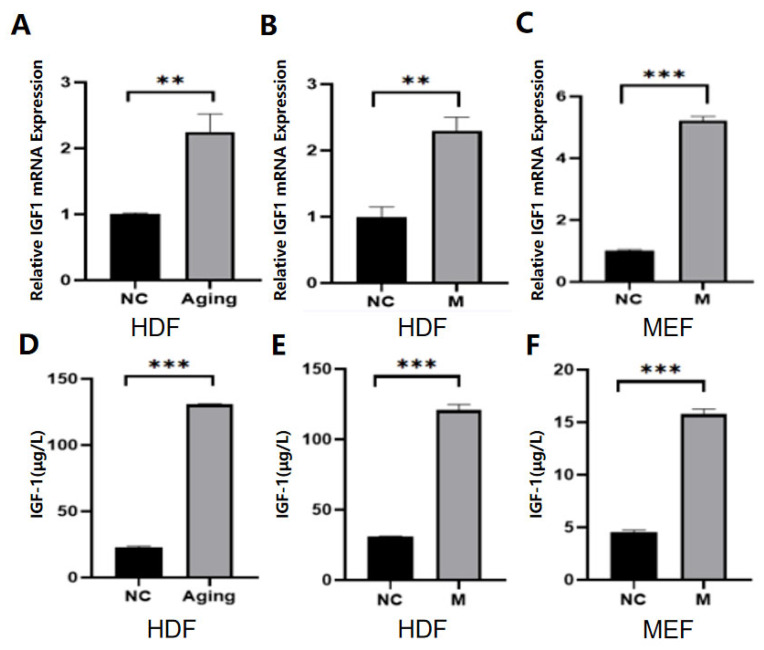
IGF-1 expression levels in normal and senescent cells. Note: NC represents the normal control group; Aging represents the replicative senescence group; and M represents the bleomycin-induced senescence group. (**A**–**C**), RT-qPCR was used to detect IGF-1 concentration in young and senescent cells. (**D**–**F**): an Enzyme-linked immunosorbent assay was used to measure the concentration of IGF-1 in young and senescent cells. ** *p* < 0.01, *** *p* < 0.001.

Three cell senescence models were established, including the HDF replication cell senescence model, bleomycin-induced HDF, and MEF cell senescence model. The successful establishment of the aging model was confirmed by cell morphology, β-galactosidase staining, RT-qPCR, and ELISA experiments for the detection of senescence-related secretory factors, and Western blot (WB) experiments for the detection of senescence-related proteins (Figure S1A–O). After the aging model was successfully established, the IGF-1 level was detected by RT-qPCR, ELISA, and WB experiments. The data showed that IGF-1 levels increased significantly with cell senescence (Figure 1A–F).

### 2.2. OA Alleviated Cell Senescence in HDF and MEF Cell Lines

**Figure 2:**
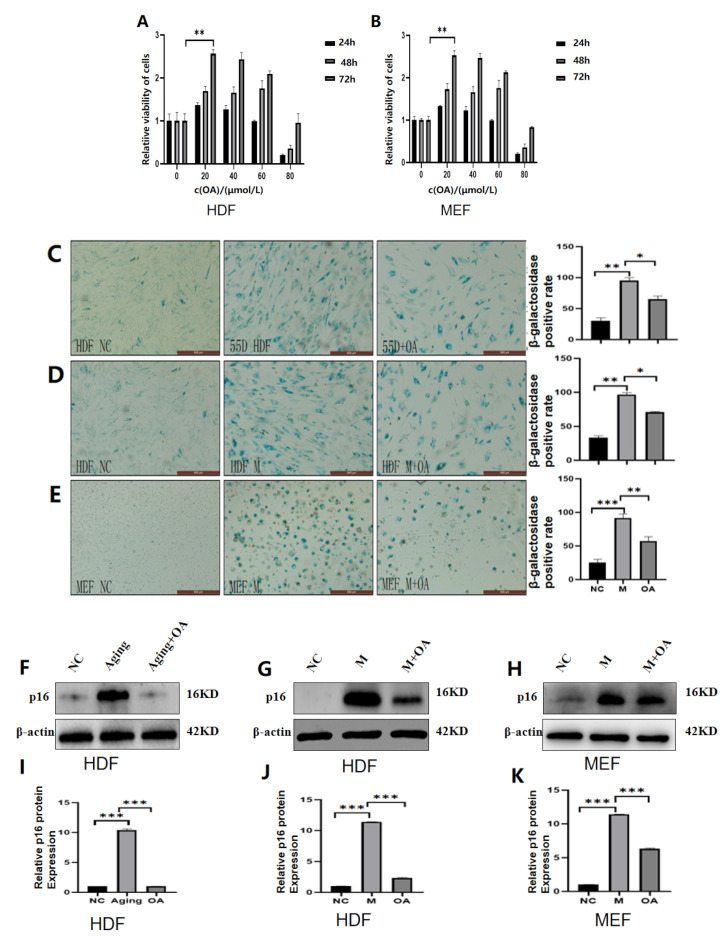

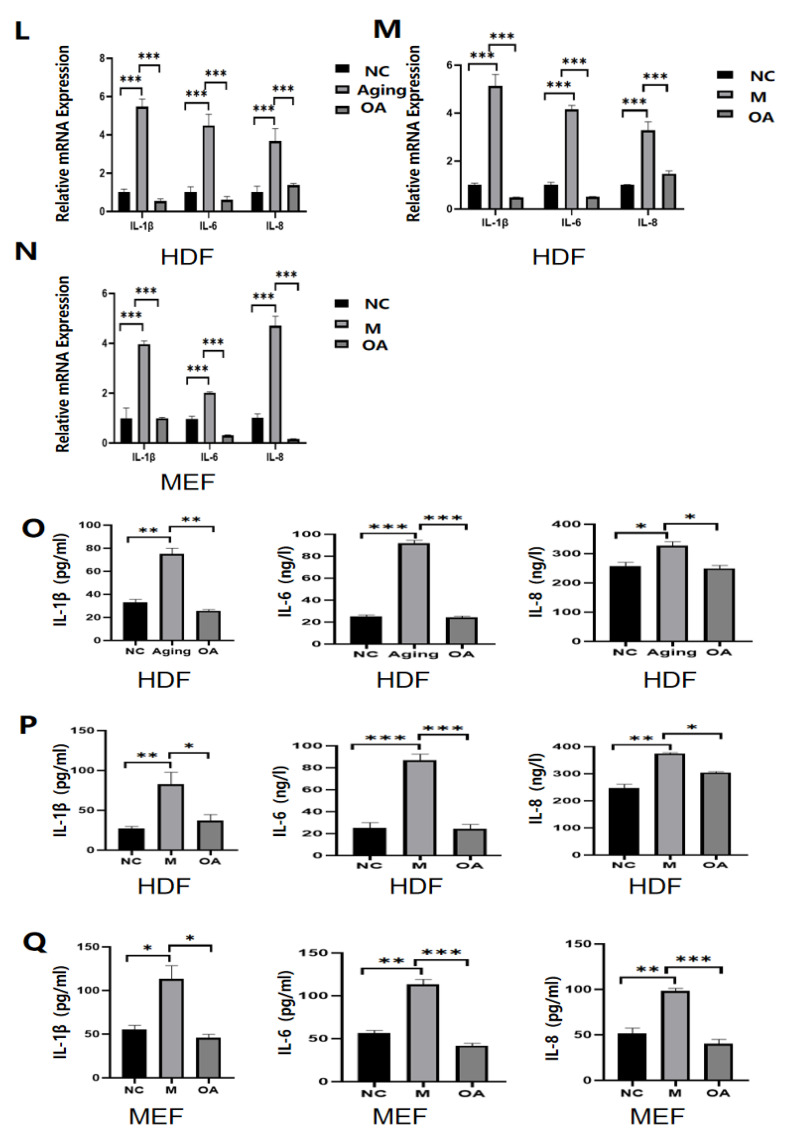
**Cell viability, expression of P16, IL-1β, IL-6, and IL-8 in the cells of OA treatment**. Note: NC represents the normal control group; Aging represents the replicative senescence group; OA represents the OA-treated group; and M represents the bleomycin-induced senescence group. (**A**,**B**) Plot of optimal concentration and time of OA on HDF cells and MEF cells, statistical results show the results of three repeated experiments. (**C**–**E**) plots of SA-β-Gal stained normal versus senescent cells and OA-treated cells, as well as statistical analysis, showing the results of triplicate experiments. (**F**–**K**) Western blot analysis and gray value analysis of senescence-related proteins P16 in normal cells, senescent cells, and OA-treated cells; the experiments were carried out in triplicate. (**L**–**Q**) RT-qPCR and ELISA experiments were used to detect the changes of senescence-related secretion factors of IL-1β, IL-6, and IL-8 in normal cells and senescent cells and after OA treatment, respectively. The experiments were conducted in triplicate. * *p* < 0.05, ** *p* < 0.01, *** *p* < 0.001.

To distinguish species-specific senescence, replicative senescence and bleomycin-induced human dermal fibroblast (HDF) cells were used. To distinguish whether senescence was species-specific, bleomycin-induced mouse embryonic fibroblast (MEF) cells were used. These senescent cells were chosen to test whether OA has an anti-aging effect. As shown in Figure 2, OA reduced the proportion of Senescence-Associated β-Galactosidase (SA-β-gal) positive stained cells in HDF cells and MEF cells and restored cell morphology with no difference among the two different sources of cell lines. Combined with the Cell Counting Kit-8 (CCK8) assay, it was concluded that the optimal concentration of OA for cell anti-aging was 20 μM and cultured for 72 h (Figure 2A,B). Western blot assay, qPCR assay, and ELISA assay were used to further verify the anti-aging effect of OA. The results showed that the p16 protein was significantly increased after cell senescence, and OA treatment significantly reduced the expression of the p16 protein. At the same time, RT-qPCR showed that OA could reduce the expression of IL-1β, IL-6, and IL-8 genes. Meanwhile, ELISA showed that OA could reduce the expression of IL-1β, IL-6, and IL-8. Our findings confirm that OA can delay cellular senescence (Figure 2C–Q).

### 2.3. OA Reduced the Expression of IGF-1 in Senescent Cells

**Figure 3:**
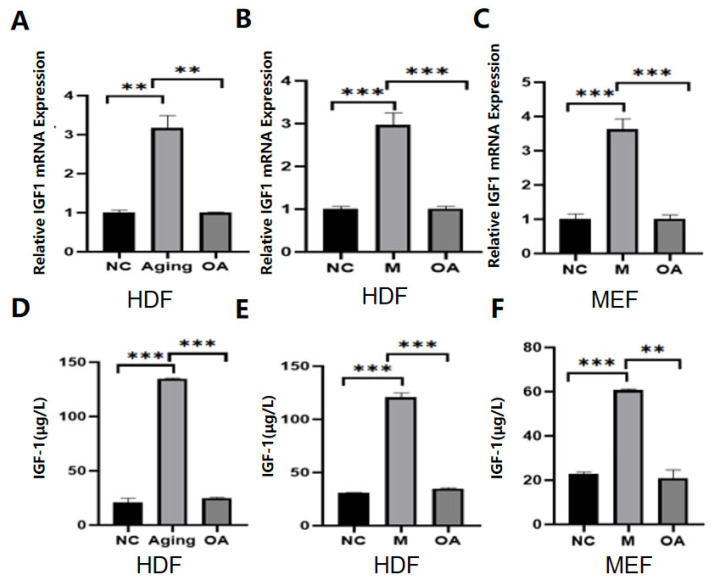
The expression of IGF-1 in senescent cells treated with OA etc. Note: NC represents the normal control group; Aging represents the replicative senescence group; OA represents the OA-treated group; and M represents the bleomycin-induced senescence group. (**A**–**C**) OA reduces IGF-1 expression in senescent cells. RT-qPCR was used to detect the changes of IGF-1 in senescent normal cells and after OA treatment. (**D**–**F**) ELISA was used to detect the changes of IGF-1 protein in normal cells after aging and OA treatment. All experiments were performed in triplicate.** *p* < 0.01, *** *p* < 0.001.

Because IGF-1 expression was increased in senescent cells per the results above, after OA treatment, the expression of GF-1 was detected by RT-qPCR (Figure 3A–C) and ELISA (Figure 3D–F), respectively. Our findings confirmed that IGF-1 expression was reduced by the addition of OA.

### 2.4. OA Delays Aging by Targeting the Expression of IGF-1

**Figure 4:**
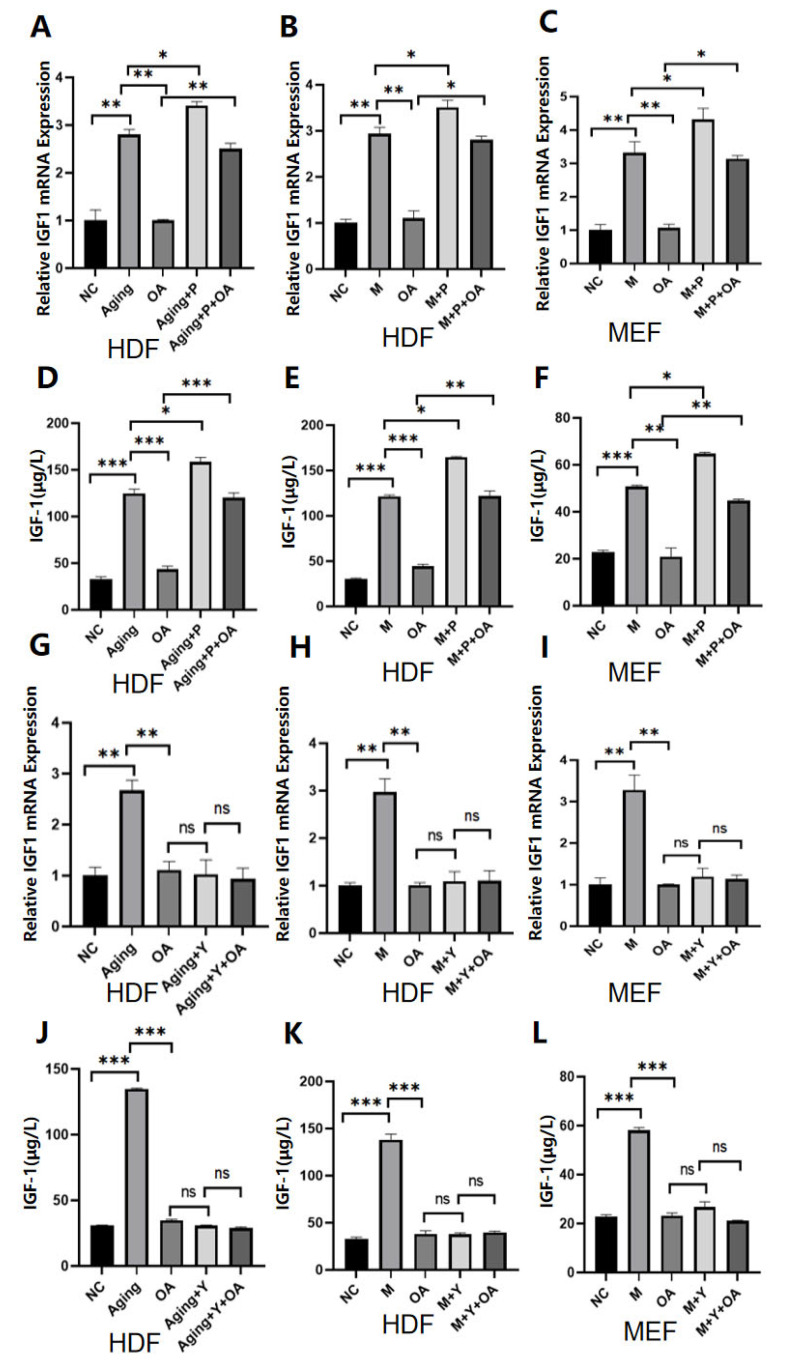
The IGF-1 expression in cells treated with OA. Note: NC represents the normal control group; M represents the bleomycin-induced senescence group; Aging represents the replicative senescence group; OA represents the OA-treated group; and P. Y represents the successful performance of the overexpression and inhibition of IGF-1. (**A**–**C**) The overexpression of IGF-1 was detected by RT-qPCR, and (**D**–**F**) the overexpression of IFG-1 was detected by ELISA. Successful inhibition of IGF-1 was detected by RT-qPCR assay in (**G**–**I**), and successful inhibition of IFG-1 was detected by ELISA assay in (**J**–**L**). The experiment was performed in triplicate. * *p* < 0.05, ** *p* < 0.01, *** *p* < 0.001.

**Figure 5:**
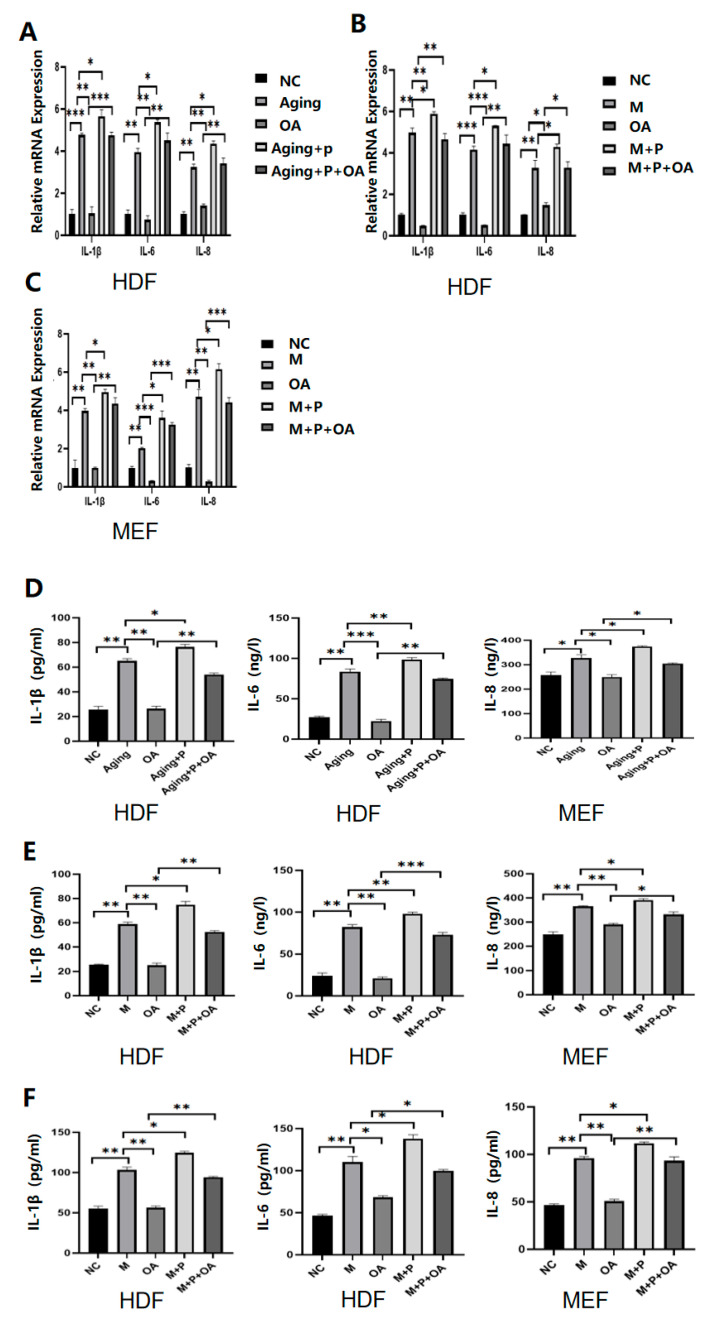

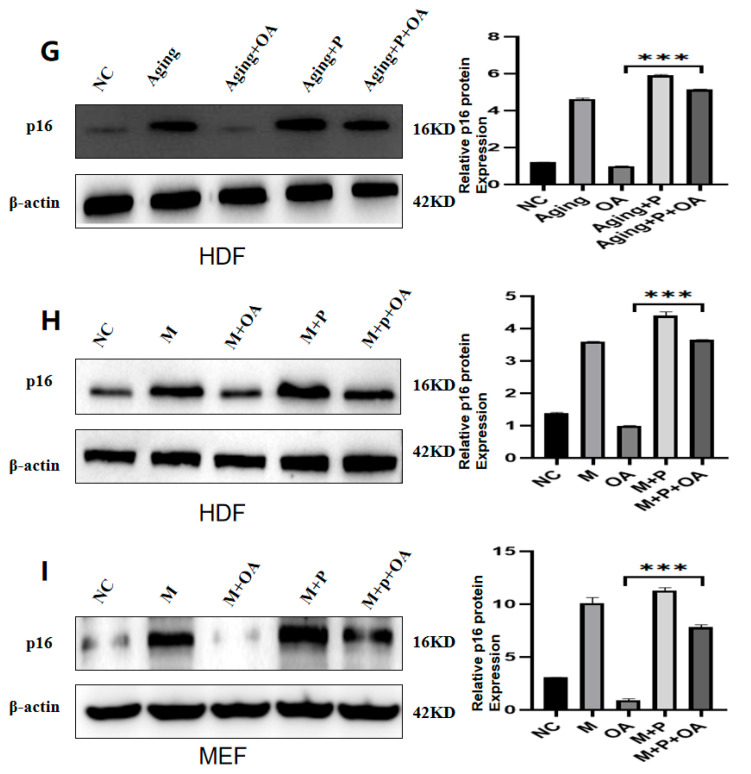
The expression of IL-1β, IL-6, IL-8, and P16 in HDF and MEF cells of different treatment groups. Note: NC represents the normal control group; M represents the bleomycin-induced senescence group; Aging represents the replicative senescence group; OA represents the OA-treated group; and *p* represents what happened after overexpression of IGF-1 and aging-related secretion factors of IL-1β, IL-6, and IL-8 were increased. The senescence-associated protein P16 was increased. (**A**–**C**) RT-qPCR was used to detect the changes of IL-1β, IL-6, and IL-8 after overexpression. (**D**–**F**) ELISA was used to detect the changes of IL-1β, IL-6, and IL-8 after overexpression. (**G**–**I**) detection of changes in P16 after overexpression, as well as gray value analysis. All experiments were performed in triplicate. * *p* < 0.05, ** *p* < 0.01, *** *p* < 0.001.

**Figure 6:**
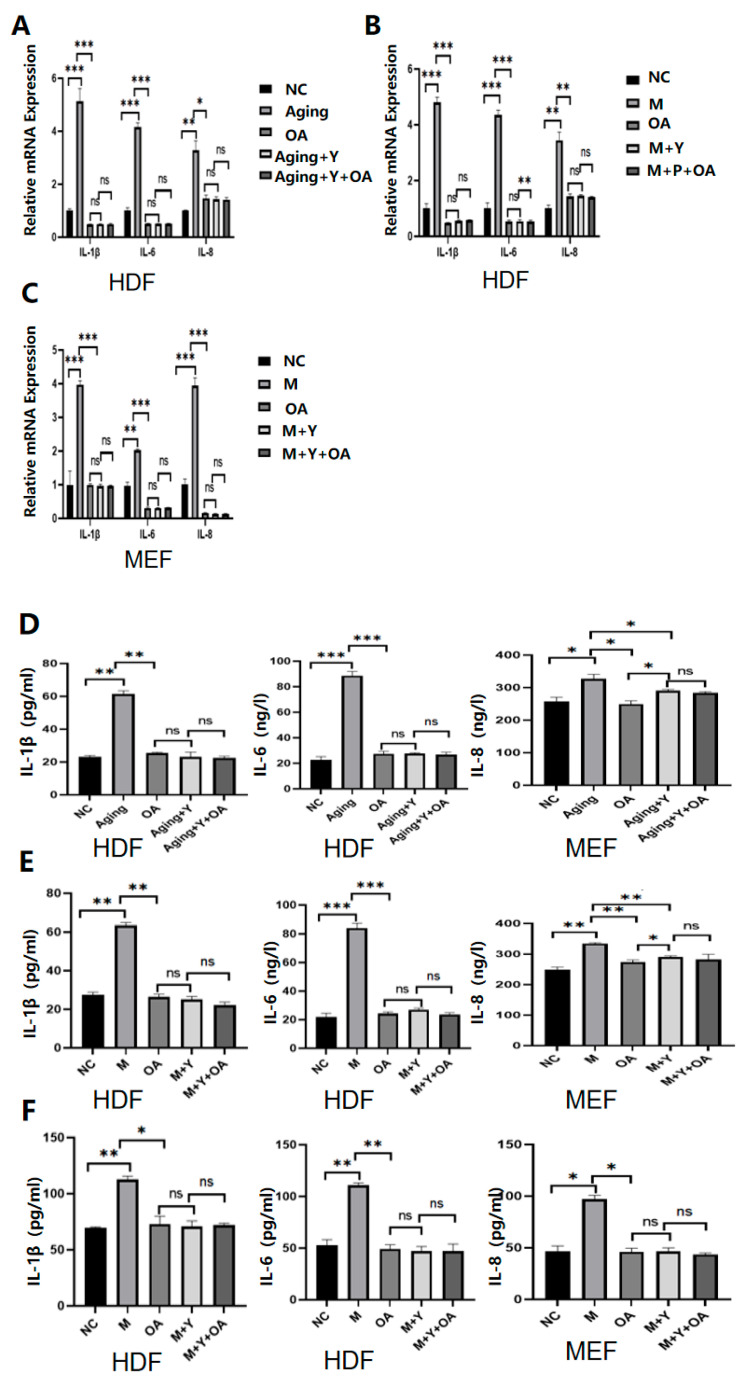

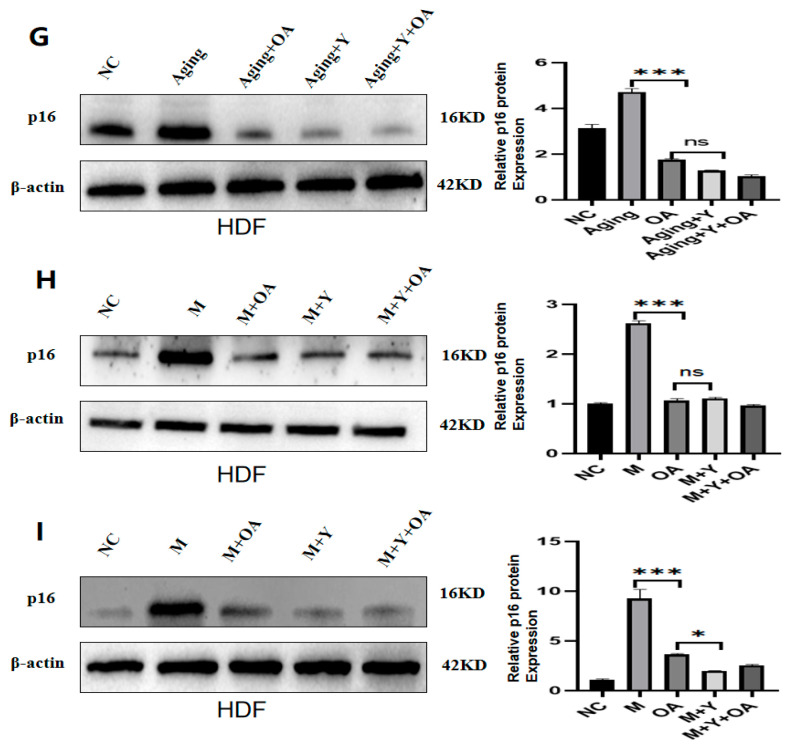
The expression of IL-1β, IL-6, IL-8, and protein P16 in different treatment groups. Note: NC represents the normal control group; M represents the bleomycin-induced senescence group; Aging represents the replicative senescence group;OA stands for OA treatment group; Y represents the IGF-1 inhibitor group, and there was no significant difference in the secretion of aging-related cytokines IL-1β, IL-6, and IL-8 after IGF-1 inhibition. There was no statistical significance in the senescence-associated protein, P16. (**A**–**C**) RT-qPCR was used to detect the changes of IL-1β, IL-6, and IL-8 after IGF-1 inhibition. (**D**–**F**) ELISA was used to detect the changes of IL-1β, IL-6, and IL-8 after IGF-1 inhibition. (**G**–**I**) The changes of IGF-1 after P16 inhibition were detected and analyzed by gray value. All experiments were repeated three times. * *p* < 0.05, ** *p* < 0.01, *** *p* < 0.001.

To determine whether OA retards aging by targeting IGF-1, HDF cells and MEF cells with increased or decreased IGF-1 expression were generated by transfection of recombinant IGF-1 protein or transfection of an IGF-1 inhibitor (Figure 4A–L). After the cells were treated with OA, the expression of IL-1β, IL-6, IL-8, and p16 protein in the cells with IGF-1 overexpression was increased compared to the control group (Figure 5A–I). There was no significant difference in the expression of IL-1β, IL-6, IL-8, and p16 protein between the IGF-1-inhibited cells and the control group after OA treatment (Figure 6A–I). These results suggest that OA delays aging by targeting the IGF-1 expression.

### 2.5. OA Delayed Aging by Inhibiting PI3K/AKT/mTOR via IGF-1

**Figure 7:**
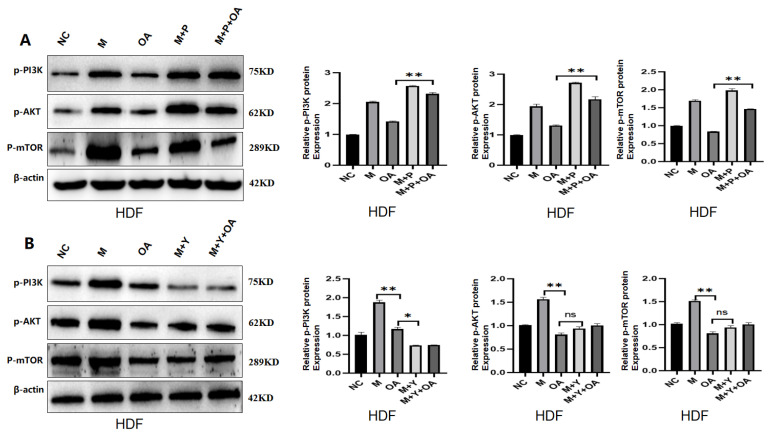
Phosphorylation levels of proteins in the PI3K/AKT/mTOR signaling pathway were altered by IGF-1 under both overexpression and inhibition conditions. Note: NC represents the normal control group; M represents the bleomycin-induced senescence group; Aging represents the replicative senescence group; OA represents the OA-treated group; and P.Y shows that the expression of the PI3K/AKT/mTOR signaling pathway was increased after IGF-1 overexpression compared with OA treatment alone. (**A**) Protein level changes in the PI3K/AKT/mTOR after IGF-1 overexpression, as well as gray value analysis. (**B**) Changes in the PI3K/AKT/mTOR protein levels after IGF-1 inhibition, as well as gray value analysis. All experiments were repeated 3 times. * *p* < 0.05, ** *p* < 0.01.

The PI3K/AKT/mTOR signaling pathway plays an important role in aging-related diseases, and there is increasing evidence that it has an important impact on longevity and aging. Based on this evidence, to further confirm the anti-aging mechanism of OA, we measured the expression of phosphorylated PI3K/AKT/mTOR proteins in the in vitro experiment. It was found that aging activated the expression of phosphorylated PI3K/AKT/mTOR, while OA inhibited its expression. We subsequently designed an IGF-1 overexpression, inhibition experiment to verify this, and the results showed that PI3K/AKT/mTOR was increased by the overexpression of IGF-1, and the PI3K/AKT/mTOR expression was decreased by IGF-1 inhibition (Figure 7A,B). These results demonstrated that OA delayed aging by inhibiting the PI3K/AKT/mTOR signaling pathway by IGF-1 in vitro.

## 3. Materials and Methods

### 3.1. Materials

OA was bought from the China Institute for Food and Drug Control (2 Tiantan Xili, Dongcheng District, Beijing, China). OA was dissolved in ethanol with added DMSO (1:1) in a final concentration (100 mM) as the stock solution. Beyotime Biotechnology provided P16 antibody (AF1672) and SDS-PAGE protein loading buffer (5X) (P0015L) (Shanghai Biyuntian Biotechnology Co., Ltd. Office address is No. 30 Xinfei Road, Songjiang District, Shanghai). Enzyme-linked immunosorbent assay kit, mouse insulin growth factor 1 (IGF-1) enzyme-linked immunosorbent assay kit (MM-0181M2), mouse interleukin-6 (IL-6) enzyme-linked immunosorbent assay kit (MM-0163M2), and mouse interleukin-8 provided by China Jiangsu Enzyme Immunoassay Industry Co., Ltd. (2nd Floor, Building 6, No.1 Wuyi Road, High-tech Zone, Dazhong Town, Dafeng District, Yancheng, China) (IL-8/CXCL8) ELISA kit (MM-0123M2), human insulin growth factor 1 (IGF-1) ELISA kit (MM-0032H2), human interleukin-6 ELISA kit (MM-0049H2), IL-8/CXCL8 ELISA kit Adsorption assay kit (MM-1558H2). Solebo supplied the mouse interleukin-1β (MouseIL-1β) ELISA kit (SEKM-0002) and the human interleukin-1β (MouseIL-1β) ELISA kit (SEKH-0002). Recombinant IGF-1 protein (Cat No. HZ-1322) was provided by proteintech Company and indicated by *p* on all figures. IGF-1 inhibitor, (CAS No.133550-18-2), provided by MCE, is indicated by Y on all notes (666 Gaoxin Avenue, Jiufeng Street, Hongshan District, Wuhan City). q-PCR primers were provided by Shanghai Sangon, and they are primer sequences (IL-6-F5′ TGGCTAAGGACCAAGACCATCCAA 3′; IL-6-R5′ AACGCACTAGGTTTGCCGAGTAGA 3′; IL-8-F 5′AGAACATCCAGAGTTTGAAGGTGAT 3′ IL-8-R 5′GTGGCTATGACTTCGGTTTGG 3′; IL-1β-F5′ CTCAACTGTGAAATGCCACC 3′; IL-1β-R 5′GAGTGATACTGCCTGCCTGA 3′; PI3K-F 5′AGTAGGCAACCGTGAAGAAAAG 3′; PI3K-R5′ GACGTGAATTGAGGTCCCTAAGA3′; AKT-F5′ TTCTATGGCGCTGAGATTGTGT 3′; AKT-R 5′GCCGTAGTCATTGTCCTCCAG 3′; mTOR-F 5′AGGTGGACCAGTGGAAACAGG 3′; mTOR-R5′ TTCAGCGATGTCTTGTGAGG 3′; IGF-1 F 5′GCTGGTGGATGCTCTTCA 3′; IGF-1-R5′TACTTCCTTCTGGGTCTTGG 3′ (No. 698, Xiangmin Road, Chedun Town, Songjiang District, Shanghai). Cell source: Peking University School of Medicine, gifted by Professor Mao Zebin. Each digestion counts as one passage.

### 3.2. Methods

#### 3.2.1. Western Blot

Western blotting was used to detect protein concentrations in HDF cells and MEF cells. Total cellular proteins were extracted using RIPA buffer (P0013B, Beyotime, Shanghai, China). Protein concentrations were calculated by the BCA method (Cat No. P0010S, Beyotime, Shanghai, China). Proteins and 2.5xSDS loading buffer were boiled together for 10 min (P0015L, Beyotime, Shanghai, China), and equal amounts of proteins (6% to 15%) were loaded on SDS-PAGE and electrophoresed first at 70 V and then gradually increased to 120 V. After gel cut and membrane transfer to PVDF membrane (Cat#IPVH00010, Immobilon-P, USA), the membranes were blocked with 5% skim milk powder and incubated with primary antibodies overnight at 4 °C. After washing with Tris-buffered saline (TBS) containing 0.1% Tween-20, secondary antibodies were incubated. A chemiluminescence imager (Tanon) was used for visualization. Band intensity was measured using ImageJ analysis software (1.54).

#### 3.2.2. Real-Time-qPCR

Total RNA extractor (Cat: B511311-0025, Sanon Biotechnology, Shanghai, China) treated cells after treatment with oleanolic acid, IGF-1 protein, and IGF-1 inhibitor. After total RNA extraction, reverse transcription was performed (Cat: D7168M, Beyotime, Shanghai, China), and the reverse transcribed cDNA was subjected to real-time fluorescence quantitative PCR (Cat: D7168M, Beyotime, Shanghai, China). Real-time PCR amplification was performed in triplicate according to the instructions of SYBR Green qPCR Master Mix (batch number: 22AE03G, ZOMANBIO, Beijing, China). Relative amounts of cDNA were determined by comparative CT using the GAPDH RNA sequence as a control. Primers were purchased from Sangon Biotechnology Co., Ltd., Shanghai, China.

#### 3.2.3. Cell Culture

Cells were provided by Professor Mao Zebin from Peking University School of Medicine, and they were Human HDF and mouse MEF cell lines. Cells were cultured with Dulbecco’s Modified Eagle’s medium (DMEM) supplemented with 10% fetal bovine serum (04-001-1ACS, Biomed, Israel), 100 units/mL penicillin, 100 mg/mL streptomycin, and 2 mM/mL L-glutamine (PMI15-0210, Procell, Wuhan, China). Passages were carried out when the cell density reached 80–90%, and one passage per digestion was counted as one growth generation.

#### 3.2.4. Cell Viability Assessment

Cell viability was detected by CCK8 assay. In 96-well cell culture dishes, 3000 cells were seeded in each well, divided into three time periods of 24, 48, and 72 h, and divided into five concentration gradients of 0 μM, 20 μM, 40 μM, 60 μM, and 80 μM. HDF and MEF cell lines were cultured in DMEM medium supplemented with 10%FBS. The cells were cultured with 0μM, 20 μM, 40 μM, 60 μM, and 80 μM of OA, and the cell viability was detected at 450 nm (24–72 h) by a microplate reader. To this end, 10 μL CCK8 reagent and 100 μL working reagent of the corresponding medium were added to each well of a 96-well culture plate. The procedure was performed for 2 h at 37 °C. For each concentration, three independent replicates were performed per well.

#### 3.2.5. Establishment of Cell Senescence Model

The cell senescence models used in the experiments included the replication-type cell senescence model and the bleomycin-induced cell senescence model. When the cell density was 80–90%, one passage was counted for each digestion until the cell growth stopped and the cell morphology changed. The expression of the p16 protein was then examined at the protein level. The expressions of IL-1β, IL-6, and IL-8 were detected by ELISA and q-PCR. SA-β-Gal staining and other senescence-related markers were used to determine the establishment of the cell senescence model. A bleomycin-induced cell senescence model was established by inducing cells with 30 mmol/mL bleomycin. On day 7 of bleomycin induction, the cells showed senescence. At the beginning of drug induction, the cells grew slowly and eventually stalled in the cycle. The expression of the p16 protein was detected at the protein level. The expressions of IL-1β, IL-6, and IL-8 were detected by ELISA and q-PCR. SA-β-Gal staining and other senescence-related markers were used to determine the establishment of the cell senescence model.

#### 3.2.6. SA-β-GAL Staining

SA-β-Gal staining biomarker activity was measured in HDF and MEF cells using the SA-β-Gal staining kit (Biotime, Shanghai, China). The cells were seeded in 24-well culture plates and cultured for 24 h, followed by bleomycin induction for 7 days and OA treatment for 3 days. Replicative senescence was induced by OA treatment for 3 days. Cells were washed once with TBS and the original medium was discarded. After the addition of fixative, cells were kept at room temperature for 15 min, washed 3 times with TBS, and SA-β-Gal reagent was added after 3 min each and incubated overnight in a 37 °C incubator. Finally, the cells were imaged under an Olympus (CKX41) light microscope, and the blue-green cells visible under the microscope were identified as senescent cells. Five fields were randomly selected, 1000 cells were examined, and the proportion of SA-β-gal-positive cells in the total cells of each group was calculated. Results are presented as the mean ± SD of three replicates.

#### 3.2.7. ELISA

Dilutions of standards were performed. Sample addition: set blank wells, standard wells, and sample wells to be tested. A sample of 50 μL was accurately added to the standards on the enzyme-labeled coated plate, and 40 μL of the sample dilution was added to the sample wells to be tested, followed by 10 μL of the sample to be tested. Incubation: Plates were sealed with a plate-sealing membrane and then incubated at 37 °C for 30 min. Preparation: 30× concentrated washing solution was diluted 30× with distilled water and then used. Wash: Carefully remove the sealing plate membrane, discard the liquid, shake dry, fill each well with washing solution, let it stand for 30 s, then discard, repeat 5 times, and pat dry. Enzyme addition: 50 μL of the enzyme-labeled reagent was added to each well, except for blank wells. Incubation: Plates were sealed with a plate-sealing membrane and then incubated at 37 °C for 30 min. Wash: Carefully remove the sealing plate membrane, discard the liquid, shake dry, fill each well with washing solution, let it stand for 30 s, then discard, repeat 5 times, and pat dry. Color development: Add 50 μL of color development agent A to each well first, then add 50 μL of color development agent B, gently shake the mixture, and incubate at 37 °C in the dark for 10 min. Termination: 50 μL of termination solution was added to each well to terminate the reaction. Determination: The blank well was set to zero, and the absorbance (OD) value of each well was measured sequentially at a wavelength of 450 nm. The assay should be performed within 15 min after the addition of the termination solution.

#### 3.2.8. Analysis of Experimental Data

All experiments in this study design were performed in triplicate. All experimental data were analyzed by GraphPad Prism software (version5.0). The *t*-test was used to compare the means of the two groups, and the analysis of variance was used to compare the two groups. * *p* ≤ 0.05, ** *p* ≤ 0.01, *** *p* ≤ 0.001, and *p* ≤ 0.05 were considered statistically significant, while NS was considered not statistically significant.

## 4. Discussion

OA is an oleanane pentocyclic triterpenoid compound widely distributed in nature. OA exists in free form and/or combined with sugar in 190 plant species from about 60 families [24]. For example, it exists in plants such as whole biloba, Hedyotis diffusa, and pristachys fructus. Oleanolic acid has many clinical pharmacological effects such as anti-inflammatory, anti-oxidation, and diuretic effects. It also has biological activities such as liver protection, anti-hyperlipidemia, anti-atherosclerosis, and anti-tumor effects. OA is an ideal drug to inhibit platelet aggregation, hepatitis type I, and chronic viral hepatitis, and has low toxicity and fewer adverse reactions [24].

Zhang et al. found that OA can prolong the lifespan and enhance the stress resistance of wild-type nematodes, and it does not act through the CR pathway, but depends on daf-16. OA regulates the nuclear localization of daf-16 and activates the transcription of its target genes, thereby promoting nematode longevity and enhancing stress resistance [25]. Peng et al. found that OA alleviates N-methyl-D-aspartate induced excitatory lung injury in mice through anti-inflammatory, anti-oxidative stress, and anti-apoptosis effects, and the mechanism may be related to activation of SIRT1 and reduction of NF-κB acetylation [26]. Duan Yulei et al. cultured primary rat Leydig cells and divided them into a normal group, model group, and oleanolic acid group. The cell senescence model and oleanolic acid group were treated with H₂O₂ (300 μmol/L) and FeSO₄ (100 μmol/L) for 2 h a day for 4 days, respectively, to establish the cell senescence model. Then, the normal group and the model group were cultured with DMEM/F12 medium containing 20 μmol/L oleanolic acid in the final concentration for 3 days, and the oleanolic acid group was cultured with DMEM/F12 medium containing 20 μmol/L oleanolic acid in final concentration for 3 days. Oleanolic acid was found to inhibit Leydig cell apoptosis and increase testosterone secretion levels, cell proliferation, EGF, and EGFR mRNA expression. The mechanism is related to the regulation of EGF and EGFR mRNA expression [27]. These results suggest that OA can delay aging.

In this study, we first established a cellular aging model and discovered that the expression of IGF-1 in aging cells was higher than that of normal cells, showing the potential role of OA in anti-aging. Furthermore, we have shown that IFG-1 can induce cellular senescence through activation of the PI3K/AKT/mTOR signaling pathway, whereas OA can significantly reduce the expression of the PI3K/AKT/mTOR, thus counteracting the pro-senescence effect. OA in this study can delay aging by decreasing the expression of these senescence-related proteins and SASP, e.g., IL-1β, IL-6, and IL-8. This research offers a new way to delay aging and provides experimental evidence for developing new anti-aging drugs.

The growth hormone (GH) axis is historically the first axis involved in the control of aging. GH acts on hepatocyte GH receptors and stimulates the secretion of IGFs. Through IGF-1R, IGF-1 activates PI3K-AKT and MTORCI networks, and thus promotes growth and development [28]. In a wide range of model organisms, spontaneous pathways or engineered mutant pathways can prolong longevity and delay the onset of age-related deterioration. Congenital defects in the growth axis led to dwarfism, but it is beneficial to the body to suppress this axis from early adulthood. Inhibition of the GH/IGF-1 pathway in adulthood and later life can prolong life expectancy in models, including mice. Inhibition of heart IGF-1R through the expression of PI3K, which is the predominant inactive p110a isoform, ultimately increases the maximal lifespan of male mice and improves heart function [29]. Moreover, the enzyme inhibition of IGF-1R by tyrosine kinases increases the antitumor immunity of cancer cells, which is required for the induction of autophagy [30]. IGF-1 plays an important role in the GH pathway and has been known to regulate aging in a wide range of organisms, including fruit flies, nematodes, and mice [31,32,33]. However, there are conflicting findings about IGF-1’s role in aging. Downregulation of the GH/IGF-1 signaling pathway in genetically modified knockdown and knockout models is generally associated with improved health in mice and reduced age-related pathologies like immunosensitivity and cancer. Furthermore, previous research has shown that the GH/IGF-1 axis is essential to repair DNA damage by modifying DNA repair genes in the early period [34,35].

The PI3K/Akt signaling pathway is an important intracellular signaling pathway involved in a variety of physiological and pathological processes. When insulin binds to its receptor, it activates PI3K, which in turn phosphorylates Akt. Activation of Akt promotes glycogen synthesis, inhibits gluconeogenesis, and regulates processes such as cell survival, proliferation, and differentiation. Studies have shown that the PI3K-Akt signaling pathway is closely related to cell senescence. Dermal-derived stem cells undergo cellular senescence in response to environmental changes, which ultimately leads to the loss of stem cell self-renewal ability. Inhibition of the PI3K-Akt signaling pathway can promote the senescence of dermal stem cells. Conversely, activating this pathway effectively inhibits senescence and promotes stem cell self-renewal [36]. mTOR, a serine/threonine kinase, is an important regulator of cell growth and p-proliferation. Studies have shown that mTOR is an important component of the oncogenic PI3K/AKT/mTOR signaling pathway and plays an important role in different basic cellular processes such as protein synthesis, cell proliferation, survival, and senescence. Studies have found that the dysregulation of the mTOR signaling pathway is closely related to a variety of diseases, including cancer, intestinal inflammation, aging, and so on [37]. Over-activated mTOR signaling will directly or indirectly induce cancer, metabolism, and aging-related diseases. Inhibition of this state can effectively delay or treat diseases caused by over-activation of mTOR and provide beneficial health effects under different pathological conditions. Therefore, mTOR is a potential target that can be used to treat a variety of diseases. There are many reasons for cell senescence, but so far it is not clear how; however, we know that reactive oxygen species, DNA damage, protein homeostasis imbalance, inflammation, etc., are related to cell senescence, and mTOR is involved in regulating these factors. Reduction of mTORC1 activity was found to significantly prolong the lifespan of Caenorhabditis elegans, revealing the role of mTOR in cellular aging.

So, does OA delay aging through IGF-1? Our study found that senescent cells are closely related to the PI3K/AKT/mTOR signaling pathway. The PI3K/AKT/mTOR is considered a key pathway for cell growth, proliferation, survival, and autophagy, interacting with a variety of proteins to form different complexes that regulate fundamental processes within cells [38]. Dysregulation of its activity is associated with a variety of diseases and disorders, and excessive activation of the PI3K/AKT/mTOR signaling pathway is detrimental to longevity and health. Our experimental results demonstrated that OA delayed aging through IGF-1 regulation of the PI3K/AKT/mTOR signaling pathway. We further verified this phenomenon by adding IGF-1 protein and IGF-1 inhibitor at the cellular level. After adding an IGF-1 inhibitor, the expression of senescence-related proteins and senescence-related secretory factors decreased. We demonstrated that this could be a potential mechanism of anti-aging in OA, and we will continue to explore this phenomenon in future studies.

Taken together, our findings suggest that food-grade OA can delay aging. These results suggest that OA is able to ameliorate and prevent cellular and physical aging to provide a theoretical basis for dietary intervention to delay aging. In fact, the functional role of OA is complex and diverse, playing different roles in different diseases. We will conduct related studies in the future to further explore the mechanism of the anti-aging effect of OA during chemotherapy. The specific mechanism needs to be further studied.

**Figure 8:**
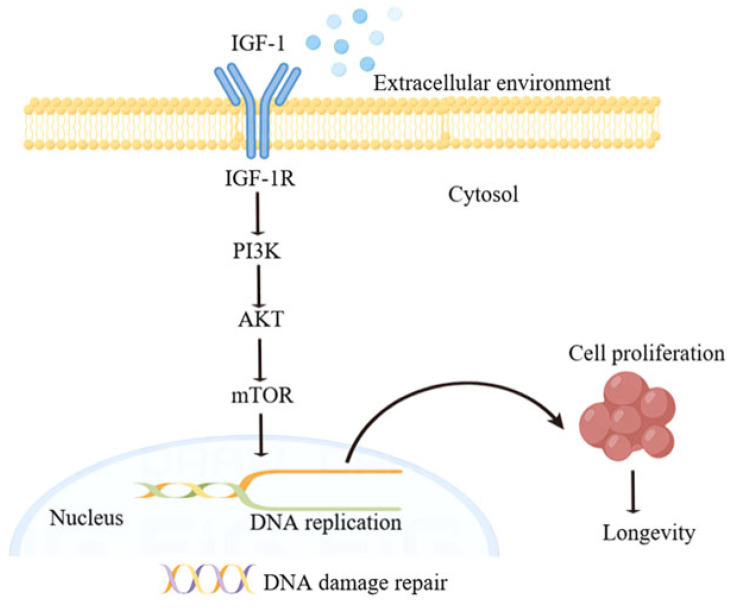
Schematic drawing of the regulatory mechanism of IGF-1 on cell aging. Insulin-like growth factor 1 (IGF-1); Insulin-like growth factor 1 receptor (IGF-1R); Phosphatidylinositol 3-kinase (PI3K); Protein Kinase B (AKT) mammalian target of rapamycin (mTOR).

This study reveals the mechanism of delaying aging in OA and explains the mechanism by which OA regulates the PI3K/AKT/mTOR signaling pathway through IGF-1. We suggest that upregulation of IGF-1 can over-activate the PI3K/AKT/mTOR pathway by binding to IGF-1R, which in turn inhibits cell proliferation and DNA synthesis (Figure 8).

## Data Availability

The original contributions presented in this study are included in the article/Supplementary Material. Further inquiries can be directed to the corresponding author(s).

## Supplementary Materials

The following supporting information can be downloaded at: https://www.mdpi.com/article/10.3390/molecules30030740/s1, Figure S1: The success chart of modeling was verified. Aging represents replicative senescence, and M repre-sents the cell model of bleomycin-induced senescence. (**A**–**C**) CCK8 assay detected cell proliferation, and cell proliferation slowed down after aging. (**D**–**F**) SA-β-Gal assay in normal and senescent cells, and respective statistical analysis. (**G**–**L**) Western blot was used to detect the expression of senes-cence-related proteins p16 (M–O) ELISA was used to detect the expression of senescence-related secretion factors IL-1β, IL-6 and IL-8. * *p* < 0.05，** *p* < 0.01, *** *p* < 0.001.
