# Supplementary material for: Oleanolic Acid Slows Down Aging Through IGF-1 Affecting the PI3K/AKT/mTOR Signaling Pathway
Source: Molecules. 2025 Feb 6;30(3):740. doi: 10.3390/molecules30030740 (PMC11820160; doi:10.3390/molecules30030740)
Supplement: Supplementary file 1 [file molecules-30-00740-s001.zip › molecules-3428586-supplementary.pdf]

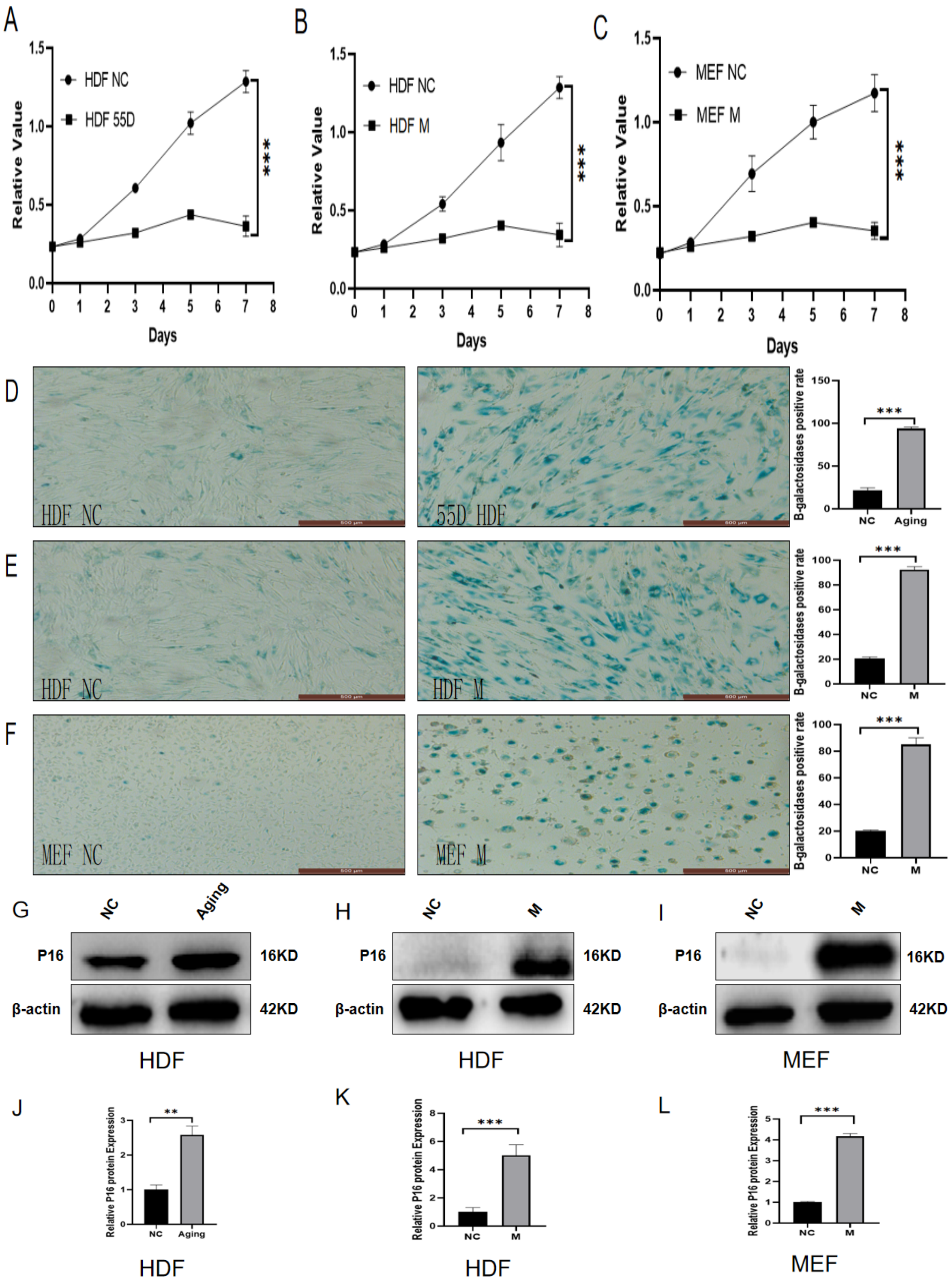

A
Relative Value
HDF NC
HDF 55D
Days
***
B
HDF NC
HDF M
C
MEF NC
MEF M
D
HDF NC
55D HDF
B-galactosidases positive rate
NC
Aging
E
HDF M
M
F
MEF NC
MEF M
G
P16
16KD
β-actin
42KD
HDF
H
I
MEF
J
Relative P16 protein Expression
**
K
L

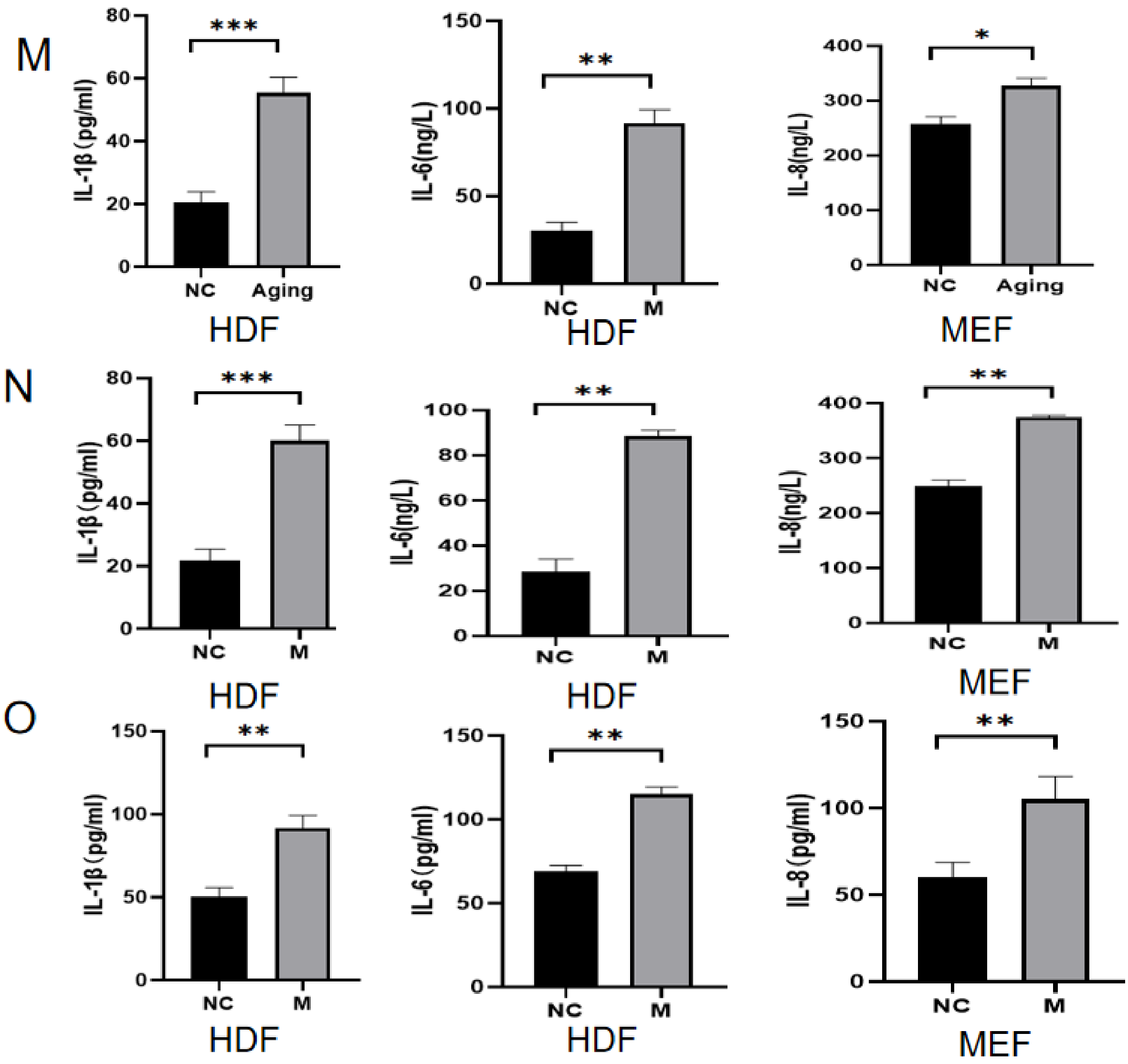

Figure S1. The success chart of modeling was verified. Aging represents replicative senescence, and M represents the cell model of bleomycin-induced senescence. (A-C)CCK8 assay detected cell proliferation, and cell proliferation slowed down after aging. (D-F) SA-β-Gal assay in normal and senescent cells, and respective statistical analysis. (G-L)Western blot was used to detect the expression of senescence-related proteins p16 (M-O) ELISA was used to detect the expression of senescence-related secretion factors IL-1β, IL-6 and IL-8.*P<0.05，**P<0.01，***P<0.001
